# Dual perspectives: Navigating the roles of physician and ethnographer in sensitive spaces

**DOI:** 10.1016/j.qrmh.2026.100048

**Published:** 2026-08-17

**Authors:** David Wenzel

**Affiliations:** School of Medical Sciences, Public Health and Epidemiology, University of Leicester, Leicester, UK

**Keywords:** Ethnography, Palliative care, Reflexivity

## Abstract

**Background:**

Positionality is central to ethnographic research because knowledge is produced through situated and relational encounters. Clinician-ethnographers occupy complex insider–outsider positions, as their professional identity may shape access, behavior, disclosure, and interpretation within the field. In this article, I explore the multiple positionalities of the physician-ethnographer in acute hospital settings and argue that professional identity should not be perceived as bias to be neutralized, but as an interpretive resource.

**Methods:**

I draw on reflexive episodes from ethnographic fieldwork examining non-invasive advanced respiratory support. I consider moments of intervention and restraint, emotional steadiness, and shifting self-presentation to examine how professional identity and authority are experienced and negotiated in practice.

**Results:**

The withholding and expression of professional authority made visible how positionality reshaped interlocutor behavior, disclosure, and the stabilization of knowledge. When I was recognized as a physician, interactions were often organized around clinical credibility and technical knowledge. When positioned as a student or outsider, interlocutors more readily articulated uncertainty, emotional labor, and the relational dimensions of care. Professional authority persisted even when unexercised, and both intervention and restraint reorganized interactions within the field. Holding multiple positionalities revealed hierarchies and moral negotiations that structure acute care.

**Conclusion:**

The physician-ethnographer’s multiple positionalities should not be understood as a methodological liability to be minimized. By reframing clinician positionality as analytically generative, I demonstrate how reflexive practice can reveal the relational and symbolic organization of clinical care. Professional identity is not external to ethnographic data, but forms part of the conditions through which knowledge is produced.

## Introduction

*The alarm blared continuously, cutting through the otherwise hushed room in Resus, an emergency department treatment area for the sickest patients coming through the front door. I stood at the edge of the cubicle, notebook in hand, watching as the nurse adjusted the tight-fitting mask on Chris* 1 *, an elderly man whose face was creased with discomfort. The machine’s alarm persisted while his oxygen levels dropped further, and Chris, confused and fatigued, was now unresponsive. The nurse, Jackie, tightened the straps further, explaining to me, “It’s his lip causing the leak.” Chris winced, but didn’t stir.*

*I clenched my jaw, my physician instincts screaming to intervene. I knew the issue was mechanical, not patient driven. I wanted to suggest repositioning the mask or flagging the need for a different interface, but I stayed silent, committed to the role of observer and unable to use my decade of clinical experience working as a palliative care registrar and general medic. I had committed to undertake an ethnographic exploration of noninvasive ventilation care because of my experiences working on a high-dependency unit during the COVID−19 pandemic. I know how patients suffer the interface, but I’ve also become adept at managing that distress.*

*Jackie leaned closer, determined, her hands adjusting the mask repeatedly as the machine’s pressure fluctuated. Chris, unconscious though he was, grimaced with the repeated tightening of the mask. At this pressure level, a wound would occur within six hours, yet the mask was still not positioned well enough to deliver the life-saving pressure that Chris required. I had not introduced myself as a doctor to this nurse, but as a PhD student. She had explained her role in detail and with the patient voice of a teacher. Would suggesting ways to improve the mask fit make her feel as if I had lied? Finally, she turned to a colleague for advice, and the mask was changed.*

*In the following minutes, Chris’s breathing stabilized, his oxygen saturation returning to a safer level. But my own tension lingered, and I wondered how my silence, or any intervention, might have changed the dynamics of care. As an ethnographer, I was there to document, not to intervene; as a physician, I couldn’t escape the impulse to act. Standing in the busy A&E where I had spent many hours treating patients, I wondered if I was morally wrong to have stepped away from clinical practice to undertake a PhD. Shouldn’t I have been the one fixing the mask?*

## A methodological reflection grounded in fieldwork

This article emerges from ethnographic fieldwork, but its focus is not to provide a sustained account of a clinical setting. Instead, it takes moments from that fieldwork as a starting point to examine what it means to inhabit the roles of physician and ethnographer simultaneously. The analysis centers on how professional identity, authority, and obligation are experienced and negotiated in practice, and how these moments of tension can be understood as analytically productive, rather than methodologically problematic. The first-person accounts that follow are therefore not presented simply as narrative reflection, but as examples through which the relational and symbolic organization of clinical care becomes visible.

I am a specialty doctor dual accrediting in palliative and general internal medicine. My training was explicitly positivist, and my engagement with ethnography and constructivist epistemology occurred only through my dual training as a clinical-academic undertaking a PhD. I was compelled to write this article following the scene described above.

The incident captured a tension that had become increasingly difficult to ignore over the course of my ethnographic fieldwork: the simultaneous presence of my clinical training and my commitment to observation, on one hand, and the uncertainty about how these roles should be enacted in practice, on the other. It was not only the question of whether to intervene, but what that hesitation revealed about my professional identity, authority, and responsibility. This article arises from that tension. It reflects an attempt to make sense of these experiences and the role they have in the organization of clinical care and my place within it.

Crucially, in examining and attempting to reconcile my multiple positionalities, I came to understand that their irreconcilability strengthens, rather than weakens, my research. My engagement with constructivist epistemology emerged from this process. This article, therefore, has two aims. The first is to examine the methodological contribution of treating multiple positionalities as an interpretive lens through which clinical practice can be understood. Second, I want to demonstrate how physicians might incorporate constructivist research paradigms to enable the exploration of phenomena normally rendered less visible by authority.

## Conceptualizing clinician ethnography

Ethnography is not simply a qualitative method, but an interpretive epistemology grounded in immersion, relationality, and the co-production of meaning within cultural contexts (Huot, 2014, Savage, 2000). In healthcare settings, ethnography has become increasingly visible, particularly in studies of complex systems and vulnerable patient groups (Black et al., 2021, Dixon-Woods, 2003, Grant et al., 2022, Savage, 2000). Unlike many clinical research traditions, ethnography does not seek detachment from the field, but instead recognizes that knowledge is produced through situated, relational encounters.

This epistemological footing generates, at times, an uneasy tension for clinician-ethnographers—especially physicians. Physicians are heavily socialized through prolonged periods of education, often straight after leaving mandatory education. This socialization involves heavy exposure to the “hidden curriculum” of medical school—an education in emotional neutralization, loss of idealism, the ritualization of professional identity, and adherence to medical hierarchies (Lempp, 2004). Physicians belong to a professional culture that prizes objectivity, decisiveness, and technical knowledge, grounded in a positivist epistemology. Ethnography, in contrast, foregrounds relational ambiguity, partiality, and reflexive engagement.

The opening vignette is not a transparent recreation of events on the ward, but a selective construction. In choosing to foreground the tightening of the mask, the sound of the alarm, and my own suspended intervention, I privilege certain relationships while leaving others in the background. My gaze renders the nurse’s hidden labor visible and positions the patient as the embodied site of technological and professional negotiation. As Calás and Smircich (1999) argue, textuality is constitutive: Writing does not simply describe the field. It participates in organizing and authorizing particular meanings. Reflexivity, therefore, must extend beyond presence in the ward to interrogate how this account itself stabilizes knowledge and authority.

Physician-ethnographers occupy a multi-dimensioned space in the field: They are insider, outsider, and, ultimately, an accountable professional. While discussions of insider–outsider dynamics are well established in qualitative research (Lichterman, 2017), relatively less attention has been given to how professional authority itself operates as a symbolic force within clinical fields. In this article, I argue that the physician-ethnographer’s multiple positionalities should not be understood as a source of bias requiring management, nor as a methodological liability to be minimized. Rather, it constitutes an interpretive resource. The shifting performances I inhabited as physician, doctoral student, and researcher did not merely affect access and rapport; they illuminated how authority shapes the relational co-production of meaning in acute care.

Drawing on reflexive episodes from ethnographic fieldwork examining non-invasive advanced respiratory support in acute hospital settings, in the following account, I explore how moments of discomfort, silence, and intervention revealed the moral and symbolic architecture of care. In doing so, I offer a reflexive methodological contribution to physician-ethnography, suggesting that holding multiple positionalities can generate analytic insight in its own right.

## The study in brief

My ethnographic study explored end-of-life care needs of patients using non-invasive advanced respiratory support (NARS), and its scope evolved directly from my clinical practice. NARS frequently complicates end-of-life care because it is used to treat common respiratory illnesses that are associated with high mortality, particularly among severely unwell patients (Aung et al., 2021, Osadnik et al., 2017). As a physician practicing at a middle-grade level, I was responsible for out-of-hours decision-making and for supporting senior colleagues during working hours. I often struggled to determine when to initiate or withdraw therapy; clinically, it is often unclear whether an acute deterioration is potentially reversible or definitively terminal. I sensed that the recognition of dying, and the often-associated withdrawal of NARS, was complex and, at times, ineffable, yet I lacked the conceptual language to fully understand how clinical judgments about hope and futility were relationally negotiated. It was this uncertainty, rather than the technology itself, that prompted my turn to ethnography.

My ethnographic study in NARS formed much of my doctoral thesis, and its results are discussed elsewhere (Wenzel et al., 2025). This article does not report the substantive findings of that study. Rather, it draws on reflexive field episodes from that work to examine how my shifting positionality as physician and ethnographer shaped and was shaped by the clinical environment. The NARS setting serves here not as an object of analysis, but as the site within which multiple positionalities became visible.

### Making the familiar strange

The challenge of “making the familiar strange” was more than methodological; it was deeply tied to my position within the field. Mentors and supervisors counseled me on the importance of engaging with what was seen, rather than what was presumed. Medical environments are deeply familiar to me; I am at home in an emergency department. That familiarity carries risk. The very ease with which I navigate clinical space can obscure its texture. The granular negotiations through which care is produced risk being flattened into what feels routine. The co-production of meaning between clinicians, patients, and technologies can disappear beneath my physician-centric reading of medical micro-politics.

In practice, this initially took the form of deliberate attempts to re-sensitize myself to the clinical environment. My strategy for this developed *a priori*. Medical staff become progressively less responsive to alarms from medical devices as the number of alarms increases (Salameh et al., 2024), and there are few places in a hospital with more alarms than the emergency department. This filtering of routine information allows for rapid decision-making. Clinicians make hundreds, or even thousands, of decisions per shift, and reducing awareness of routine information helps to maintain decision-making capacity (Zheng et al., 2018).

My plan was therefore to attend deliberately to the noise of alarms: to identify which machines produced them, to follow their rhythms, and to force them back into my perceptual field. To further unsettle familiarity, I began each period of observation by consciously smelling the ward. After years of clinical practice, I have learned not to register hospital smells. This strategy was informed by the work of Pink (2015), who argues that sensory data is an important component of ethnographic data.

In practice, however, re-sensitizing myself to alarms and smells proved nearly impossible. This failure marked an important shift in how I understood the problem. Each time I focused on a single alarm, several others sounded elsewhere. The hospital smelled strongly of chlorine-based cleaning agents, and within minutes, I became nose-blind. My *a priori* strategies failed to make the familiar strange. More importantly, they failed because I was attempting to unsettle familiarity at the level of sensation alone. I was failing to engage with my positionality and how it influenced my professional behavior and authority. I treated sounds and smells as stable environmental artifacts—features that existed independently of interpretation and could simply be “noticed” more carefully. In doing so, I underestimated how deeply professional training organizes perception itself. What I had taken to be sensory dulling was not merely inattention; it was a cultivated way of seeing and not seeing.

A more revealing shift occurred not through sensory strategies, but through a change in how I was positioned within the field. This unexpectedly more effective method of rendering the familiar strange came in a more epistemologically congruent way that naturally evolved over the course of the study: entering the clinical environment with a student lanyard. As a medical registrar working across multiple wards, whichever ward I enter, there is always someone waiting to ask me a question or assign me a task, making my arrival noted. Often, I am accompanied by or travel with a small group of junior staff, medical students, or attending physicians. I am recognized as a decision maker. My presence, my scrubs, my stethoscope, and lanyard all carry symbolic weight and authority.

Arriving at the ward with a student lanyard rendered me almost completely invisible. Not only was my customary welcoming committee absent, I found staff who did inadvertently make eye contact with me quickly dropped their gaze down to my lanyard, before promptly looking away. I was no longer hailed as “doctor,” but absorbed into the background of the clinical space. No longer welcomed into the throng of medical staff, my usual role in the construction of clinical meaning was disrupted. I was no longer standing in the steady stream of medical information through which decisions are narrated, justified, and stabilized. The absence of my welcome felt strange. It induced a heightened awareness of what was said, who said it, and who was authorized to respond. Removed from the central flow of physician dialogue, I became more attuned to forms of labor that I had previously overlooked, particularly the emotional and relational work of nursing staff.

What this revealed was not simply a change in access, but a reconfiguration of how meaning was produced around me. By becoming socially defamiliarized, I began to inhabit what Gunderson (2020) describes as a reflexive stance. This stance was not achieved by simply sharpening my perception; it was achieved by unsettling my professional identity in ways I had not anticipated.

### Psychological safety in the field

Psychological safety is extensively considered within the ethnographic community, and, particularly when researchers work within their own cultural background, there is a material risk of losing one’s “cloak of academic objectivity” (Behar, 1996, p.11). Emotional detachment can masquerade as rigor, but denying or suppressing emotion detracts from, rather than enhances, ethnographic data. When emotionally charged encounters are bracketed or avoided, the relational dynamics through which meaning is produced are flattened (Davies & Spencer, 2010).

This tension between emotional detachment and engagement was not abstract, but shaped by my clinical training. For much of my medical training, emotional neutralization was not formally taught, but implicitly modeled. This is a key feature of the hidden curriculum (Lempp, 2004). As a medical student, I witnessed a patient request euthanasia early in my training. She was an older woman with metastatic pancreatic cancer in severe, unrelieved pain. At the end of the ward round, the attending, altogether too cheerful and upbeat, asked if there was anything else he could do for her. She replied, “Please doctor, just kill me. I can’t take this pain.” The attending answered quickly, “Well, you know I can’t do that,” and moved on without further comment When I later asked him about it, he said, “Well, it’s unusual for patients to ask you to bump them off, but it happens.” The message was clear: We don’t talk about these things. Such moments trained me to compartmentalize emotion and to interpret profound expressions of suffering as routine clinical interactions. This training did not disappear when I entered the field as an ethnographer. It shaped not only how I responded to suffering, but what I registered as noteworthy.

Over time, these practices became rationalized within my clinical work. As a more senior trainee, I came to rationalize these habits. I cannot, or perhaps should not, carry one patient’s emotions into the next encounter. I cleanse my thoughts as I wash my hands so that each patient may face me from what feels like a neutral standpoint. This ritual—mundane, yet deliberate—sustains the performance of objectivity, even when emotional residue persists.

These differences in how emotion is understood became particularly visible in the design of the study. In developing the study protocol, considerable attention was given to my emotional well-being, largely by others. I, like many physicians, tend to define myself by resilience, often coupled with limited regard for self-care (Aronsson et al., 2000, Montgomery, 2014). The study presented several significant risk factors for psychological and moral injury. “Psychological injury,” in this context, refers to the emotional or cognitive consequences of a traumatic event (Feriante & Sharma, 2023) such as witnessing distressing clinical encounters. “Moral injury” refers to the distress caused by actions or inactions that violate one’s moral compass, such as being complicit in care perceived as harmful or poor quality (Koenig & Al Zaben, 2021).

In this article, I use “moral injury” to describe the individual distress I experienced when my actions appeared to conflict with my beliefs about what good clinical care required. However, this distress cannot be understood as purely personal. My beliefs about responsibility, harm, and intervention have been shaped by professional normativity and by the cultural expectations of medical practice. Moral injury, therefore, emerges from the mutual constitution of personal morality and professional socialization. It is experienced individually, but produced through a moral world in which physicians are trained to recognize suffering, assume responsibility, and act.

My clinical supervisor, a physician exposed to the same socialized learning, had little to no concern about my psychological wellbeing in the field. My academic supervisor, however, who has an anthropological background, expressed a degree of concern. She highlighted my limited consideration of self-care in the planning stages and ensured that psychological supervision, regular debriefs, and opportunities to pause data collection were formally embedded in the research design. The divergence between their responses reflected more than personal disposition; it revealed disciplinary differences in how emotional exposure is understood and managed.

In practice, I found that my emotional neutralization functioned as a form of protection in the field. I was privileged during this research to join families and physicians discussing the withdrawal of treatment due to its failure. I was able to watch these interactions and remain present, rather than being consumed by the intensity of grief and emotion. I was able to attend to gestures, pauses, shifts in tone, and the relational work of healthcare, rather than being overcome.

This steadiness, however, was not simply protective, but analytically productive. My relative emotional steadiness during fieldwork did not diminish the depth of my observations. I was not overwhelmed by the intensity of the encounters I witnessed, nor did I feel compelled to distance myself from them in order to cope. Because I did not need to protect myself from the emotional force of these moments, I could remain present within them. This steadiness allowed me to attend closely to how emotion moved through the room: who was responsible for containing it, who was permitted to express it, and how it shaped decisions. In this sense, emotional regulation was not simply a coping mechanism, but a form of professionally cultivated affective labor, shaping my presence in the field and my capacity to recognize the less visible emotional labor through which care was sustained.

That is not to say that I was immune to moral injury or that my psychological well-being did not influence my placement within the field. This became particularly apparent during arterial blood gas sampling, a procedure that frequently informed decisions about NARS, but was technically challenging and exceptionally painful for patients. Because these samples were central to patients’ clinical trajectories and often placed considerable pressure on staff, I witnessed dozens of attempts to obtain them.

During my ethnographic fieldwork, one encounter remains vivid. A junior nurse undergoing the necessary training to collect gas samples was being supervised by a senior nursing colleague. As I watched the nurse approach the patient, I could immediately tell they would not succeed in obtaining the blood gas. The angle of approach was too shallow, the needle moved too fast, and the anatomical position of entry was incorrect. The junior nurse tried twice before the senior took over, who then also missed twice. The blood gas was eventually obtained by a physician 40 minutes later.

During many such attempts, I noticed myself clenching my jaw and holding my breath. I experienced a visceral frustration at not intervening. I felt, perhaps arrogantly, confident that I could have obtained the sample on the first attempt. What unsettled me was not simply the patient’s pain, but the suspension of my own competence. I was present as a doctor who would not act.

Over time, I found I could no longer watch these procedures. I began stepping away during attempts and returning once the sample was placed in the analyzer. This was not only a practical adjustment. It was a recognition that observing repeated failed attempts produced a form of moral injury for me. I felt unexpected guilt that I was withholding my expertise to sustain my role as researcher. I experienced myself as standing at the boundary of my professional obligations, witnessing harm I believed I could mitigate and choosing not to intervene in order to gather data.

In retrospect, this tension was not only a personal response, but an expression of professional authority. The authority I carry as a doctor clinically did not just allow me to act, but compelled and obligated me to. Resisting the urge and moral imperative to intervene cost me moral injury.

As a person, I adjusted my exposure to certain components of the field. As an ethnographer, however, I returned to these moments reflexively, not to justify or resolve them, but to examine how my multiple positionalities shaped where I stood, what I witnessed, and what I felt compelled to do.

### Transmitting positionality

Positionality is co-constructed. I entered the field with a personal and professional identity shaped by medicine, and interlocutors responded by interpreting and assigning meaning to that identity, which was constantly renegotiated (Taylor, 2011). My positioning was not solely mine to declare; it was relationally negotiated in each encounter. While my background afforded me significant insider status, I simultaneously inhabited aspects of outsiderhood. I was, at minimum, living in the hyphen between insider and outsider. This status is important; it gave me access to one of the most protected spaces in medical culture: the bedside of dying patients (Lawler, 1991). But it also gave me the authority of a physician—authority that, although less dominant than it once was still influences how healthcare teams work today (Crampton et al., 2023). My presence had the potential to reorganize the interactions I sought to explore.

The fieldwork was conducted in a hospital where I had previously worked clinically. Part of the rationale for this was the opportunity to leverage pre-existing social relationships to facilitate access. I was cognizant of the landscape of the field, making trust and rapport quicker to establish (Roseneil, 1993). However, this created further possibility that my presence reorganized clinical interactions. Awareness of how my presence may influence the behavior of interlocutors (Taylor, 2011) became central to my reflexive practice. Subtle shifts in how I introduced myself, how I dressed, and how I responded to clinical discussions altered the assumptions interlocutors made about me. Positionality thus emerged not as a static methodological category, but as an evolving, relational phenomenon, continuously negotiated and consequential for what became visible in the field.

Over the course of fieldwork, I became increasingly aware that how I declared, or withheld, elements of my professional identity altered the texture of interaction. Rather than occupying a fixed role, my self-presentation shifted across encounters. These shifts revealed patterned differences in how interlocutors responded, what they disclosed, and how authority was negotiated. In the following section, I examine how these shifting positionalities operated within interactions with clinical staff.

### Positionality and professional authority in staff interactions

Broadly, these variations in positionality clustered into three recurring modes of self presentation:

• The Insider: scrubs with student lanyard, introduced self as a doctor doing a PhD, free use of medical knowledge.
• The Hyphen: scrubs with student lanyard, introduced as a PhD student, free use of medical knowledge.
• The Outsider: trousers and shirt, student lanyard, introduced as a PhD student, no expression of medical knowledge.

The extent to which I presented or foregrounded my professional identity appeared to meaningfully change the way interlocutors interacted with me in the field.

When I entered the field as an Insider, my access to patients was virtually unrestricted. Introducing myself as a palliative registrar conducting research was met with warm greetings. Members of staff trusted me implicitly; indeed, on multiple occasions, I needed to prompt professional consultees to properly review the declarations they signed. Rapport was often immediate, facilitated by clinical credibility and pre-existing relationships. Yet this familiarity came with consequences.

These interactions revealed not simply differences in access, but differences in how professional authority shaped communication. Presenting as an Insider led staff to quickly share explicit inter-professional disagreements and readily included me in informal gatherings, even inviting me to the staff Christmas party. However, clinical interactions tended to become more formal “case presentations,” often stripped of emotional reflections or deeper personal insights. Interlocutors seemed to shape their dialogue toward my perceived professional expectations, sometimes making my data feel thin. Open-ended questioning could partially offset this, but many interlocutors remained reluctant to share the moral and relational aspects of their work.

In contrast, altering my self-presentation disrupted these assumptions and revealed different interactional dynamics. As a Hyphen or Outsider, I often found initial interactions cool or cautious, requiring more effort to build trust and credibility. Staff greeted me politely, but withheld immediate familiarity. These early moments of entry proved consequential. Without the immediate authority of “physician,” I was positioned closer to learner than decision-maker. Nursing staff, in particular, exercised greater gatekeeping around patient access, sometimes insisting that physicians confirm that my presence was acceptable. Authority was no longer presumed; it was negotiated. This welcome was reminiscent of my experiences as a medical student.

Under these conditions, staff took on a gentler explanatory tone. They shared their clinical assessments, but also their reasoning, hesitations, and emotional responses in greater detail. These conversations made forms of emotional and hidden labor easier to identify, including the reassurance of patients, the management of uncertainty, and the informal negotiations through which care was coordinated. However, when my questions revealed clinical fluency, interlocutors frequently sought clarification of my background. Once my medical identity was confirmed, the interaction often recalibrated. The tone shifted back toward insider discourse: compressed, technical, and less reflective.

When I used the Outsider approach, wearing trousers and a shirt with a student lanyard, this made interlocutors, especially healthcare assistants, mistake me for a senior doctor. The semiotics of dress proved unstable, and the identity that I perceived I presented was often understood differently by interlocutors. I quickly reverted to scrubs to be more in keeping with the way medical students dress in the hospital. Interacting thereafter as a student was similar to the experience of the Hyphen approach. However, attempting to conceal my medical knowledge was challenging and made it more difficult to elicit information about interlocutors’ perceptions of patient care.

For example, a junior doctor explained to me that a patient was on NARS for “lung disease.” When I asked what lung disease (as this contextualizes the mortality rate), he further explained, “He used to smoke, so he has lung scarring.” I was unable to find out from this interaction if the patient had interstitial lung disease or chronic obstructive pulmonary disease (COPD). The range of mortality for the two conditions is broad, and I wanted to explore the team’s perspectives on prognosis, but struggled to do so without fully understanding the clinical context. I spent much of the following hour frustratedly trying to guess the diagnosis from other interactions. (It was COPD.)

These variations did not point to an optimal positional stance, but to the underlying organization of authority within clinical practice. There was no “best” positional stance, as perhaps I had naively hoped to generate. Rather, they exposed how authority, credibility, and disclosure are socially organized within acute care. My shifting presentations did not merely alter access; they illuminated hierarchies that structure who is expected to speak, who is expected to listen, and who is permitted uncertainty.

The Hyphen mode proved the most sustainable over time, not because it was optimal, but because it held tension rather than resolving it. In this positioning, I felt less as though interlocutors experienced my presence as evaluative. Unexpected disclosure of my medical background did not typically produce offense, though a small number of participants became visibly less comfortable with my continued observation once my clinical identity was confirmed.

At times, my positioning was disrupted by others. Staff would jokingly refer to me as “Boss” or “Imposter,” collapsing the ambiguity I was attempting to maintain. I worried about the ethics of partial disclosure and the possibility of perceived deception. Yet these moments were often met with humor rather than hostility. The instability of my identity was not simply a methodological inconvenience; it revealed how strongly the category of “physician” organizes interaction.

### Positionality and disclosure in patient and family encounters

While these dynamics were most visible in interactions with staff, my positioning operated differently in encounters with patients and their families. I did not disclose my medical background to patients or relatives. This decision was carefully considered during ethical planning. The intention was twofold: to avoid blurring the boundaries between research and clinical care and to minimize the risk that patients or families might feel coerced into participation by the authority associated with the physician role.

In practice, patients and their families were overwhelmingly kind, taking a more pastoral role than I had expected. They were keen to include me in discussions to secure both my learning and the integrity of the research. They recounted personal histories, described their fears about deterioration, and reflected on the care they were receiving. Notably, none asked directly about my professional background. I would not have concealed it had they done so.

Yet, the absence of declared medical authority did not necessarily equate with the absence of authority altogether. My presence within clinical space, proximity to the medical team, and embodied familiarity with hospital routines might still have positioned me in ways that shaped disclosure. Here too, multiple positionalities are not erased, but reconfigured.

Without the visible markers of physician authority, my interactions with non-clinical interlocutors unfolded differently from those with staff. I was not positioned as evaluator or decision maker, but as witness. What emerged were narratives centered not on clinical reasoning, but on meaning: how illness disrupted family roles, how hope was recalibrated, and how suffering and tolerance were interpreted. The absence of declared medical authority appeared to create space for forms of disclosure that were less constrained by hierarchy.

### Handling error without jeopardizing access

Throughout fieldwork, I was repeatedly confronted with moments where clinical error or suboptimal care became visible to me. As a physician, these moments carried implicit obligation. As an ethnographer, they required restraint. The boundary between observation and intervention was neither fixed nor neutral; it was negotiated in real time. These moments are more than ethical dilemmas; they are challenges through which the operation of professional authority became visible.

My clinical training grants me the authority to deliver care in all areas of my fieldwork. During the design stage of the research, a rigorous, ethical framework was created to navigate which medical errors and incidents obligated me to leave my ethnographer role and assume a physician position. It was unthinkable to an ethics panel and to my own professional identity that there would never arise a situation requiring me to step out of the ethnographer’s role and assume that of physician.

Medical error exists on a spectrum of severity from no-harm/near miss to catastrophic (Rodziewicz et al., 2025). While the impact of error may be codified, what constitutes error is socially interpreted and hierarchically negotiated. This is especially true around issues of poor practice. Judgments about what counts as substandard care are heavily influenced by professional authority and social norms. As a physician, trained in palliative care, I carry significant knowledge and power and often find myself in senior roles in determining the quality of care. Yet in the field, exercising that evaluative authority would have fundamentally reshaped the interactions I sought to observe. To attend to hidden labor, relational care, and the emotional texture of decision-making, I had to resist becoming the arbiter of error.

Instead, the focus was on when to intervene on medical events that are almost universally constructed as error, those that demanded ethical action. Certain scenarios, such as cardiac arrest without available medical support or unequivocal medication errors involving wrong drug, wrong dose, or wrong patient, would have demanded immediate intervention. These instances sit at the far end of the spectrum, where professional obligation is difficult to contest. Most situations, however, occupied a far more contested space: clinically debatable and hierarchically mediated.

The following episode illustrates how these tensions unfolded in practice: On the first day of fieldwork, within a half hour of arriving, I witnessed my first apparent medical error. A nurse in the emergency department escalated a patient in bed six with a blood sugar of 1.6 mmol/l (critically low). The unit’s senior physician responded promptly, but attended the wrong patient (whom I was observing in bed eight) to perform an emergency blood sample to assess the blood sugar level. Because of the use of ventilatory support, the physician elected to check the blood sugar with an arterial sample. The patient did not need this test at the time, and arterial sampling is known to be exceptionally painful (Matheson et al., 2014).

I recognized the discrepancy immediately. The escalation had been for bed six; the physician was now preparing to perform an invasive procedure on bed eight. My instinct was to speak. The error was straightforward, the correction simple. Yet the dynamics of the moment were not. This was a senior doctor responding swiftly to an emergency call. To intervene publicly would have meant interrupting authority in front of nursing staff and the patient. I found myself calculating not only clinical risk, but relational consequence.

I decided to intervene. The clinical stakes were clear: One patient risked unnecessary pain. Another risked delayed treatment. I spoke quietly, clarifying which bed had triggered the escalation. The physician paused, reassessed, and immediately redirected attention to the correct patient. The physician appeared embarrassed. After stabilizing the hypoglycemic patient in bed six, the physician returned to bed eight and explained how the confusion had occurred.

What followed was less an apology than an accounting. The physician described the cognitive load of managing multiple simultaneous escalations, the spatial disorientation of identical bed spaces, and the relentless tempo of the emergency department. The error, while technically simple, was situated within systemic pressures. My intervention did not fracture rapport as I had feared. Instead, it opened a conversation about the demands placed on clinicians and the thin margins within which they operate.

The moment of intervention shifted me from observer back into the role of physician, reactivating the authority embedded in my professional identity. Silence would have preserved my observational stance, but at potential clinical cost. The physician’s subsequent account of the error was neither defensive nor apologetic; instead, it became a relational explanation of how care unfolds under pressure. Through that exchange, the boundary between doctor and ethnographer was not dissolved, but renegotiated. My roles were not sequential, but continuously stabilized through interaction.

A contrasting form of tension emerged in situations where intervention was withheld. Later in the fieldwork, I followed a particularly complex case in which NARS had been initiated despite questionable clinical indication. The patient was terminally agitated, and the palliative care team were titrating medications for symptom control. In my clinical judgment, the patient appeared significantly underdosed, and I believed that a substantial increase in background medication was warranted.

A specialist nurse reviewed the patient and proposed a modest dose escalation. The nurse then turned to me and asked for my view. The request immediately unsettled the boundary I had been attempting to maintain. I was not present as a member of the clinical team, yet my silence could be read as withholding expertise. I judged this situation to be one of clinical interpretation rather than unequivocal error and declined to offer direct advice.

The nurse responded, “So, you do know, but you won’t tell me.” This was not an unfair assessment, and I replied, “I’m not indemnified to provide medical opinions in this role, but this would be a reasonable thing for you to check with a senior.” The exchange ended there. In subsequent encounters, however, the nurse occasionally referred to me as “just a student,” emphasizing the boundary I had insisted upon.

This interaction revealed a different dimension of holding multiple positionalities. In refusing to activate my clinical authority, I did not remain neutral; I was reclassified. My restraint was interpreted not as methodological necessity, but as withdrawal of collegial support. The boundary between ethnographer and physician was maintained, but at relational cost. Unlike the earlier episode, where intervention stabilized hierarchy, here non-intervention destabilized it.

Taken together, these episodes demonstrate that authority is not only exercised through intervention, but also through its deliberate restraint. In both intervention and restraint, my professional identity remained active and my positionality continuously negotiated. Whether exercised or withheld, my authority reorganized interactions, and in specific circumstances, my authority compelled action. These moments underscored that holding multiple positionalities is not managed once and resolved; it is continuously enacted. My professional identity and authority, even when restrained or concealed, remain present.

## Conclusion

The physician-ethnographer does not move between discrete roles of physician and researcher. Rather, these identities coexist and, at times, collide. Their boundaries are continuously renegotiated in practice. Throughout this fieldwork, moments of intervention and restraint revealed that professional authority does not disappear when unexercised. Whether enacted or withheld, it reorganized interaction and shaped disclosure. Interpretations of error, care, and responsibility were structured not only by individual judgment, but by the broader moral and hierarchical organization of clinical practice.

I argue that holding multiple positionalities, therefore, is not a methodological liability to be neutralized. It is an interpretive resource that can be mobilized to explore hidden labor and the relational dimensions of care. The shifting performances of Insider, Hyphen, and Outsider did not simply affect access; they revealed how authority shaped what could be said, by whom, and in what form. When I was recognized as a physician, knowledge was often compressed into technical shorthand and organized around presumed clinical competence. When I was positioned as a student or outsider, other forms of knowledge became more available, including hesitation, uncertainty, emotional labor, and relational care. Emotional modulation, professional restraint, and ethical tension were therefore not contaminants of the data, but part of the ethnographic material itself.

This argument contributes to methodological debates on reflexivity by moving beyond the acknowledgement of bias or situatedness. Reflexivity, here, is not a defensive statement of limitation, but a mode of analysis. Attending to the instability of my own position allowed me to examine how professional identity structured the field itself: who was authorized to speak, who was expected to explain, who could disclose uncertainty, and when clinical responsibility demanded action. In this sense, the physician-ethnographer’s authority is not external to the data; it is part of the relational conditions through which data are produced.

For medical practitioners more broadly, these findings invite reflection on how professional authority is experienced by others in the clinical environment. Authority not only enables decision-making, but structures who is heard, what is said, and how care is negotiated. Attending to these dynamics may make visible forms of labor—particularly relational and interpretive work—that are often overlooked within biomedical models of practice.

For future research into medical practice, this work suggests the value of approaches that foreground reflexivity and positionality as integral to, rather than in tension with, rigorous inquiry. Rather than seeking to bracket professional identity, studies of clinical work may benefit from examining how that identity shapes what can be known, seen, and said. In doing so, clinician-ethnography can offer a distinctive contribution to understanding the complexity of contemporary healthcare beyond what is accessible through more positivist methodologies alone.

## Ethical approval

Specific ethical approval was not required for this article. However, the study referenced therein was given by National Health Service Research and Ethics Committee Wales REC7on the 2nd of August 2024 (IRAS ID 339785, REC reference 24/WA/0215). Participants with capacity gave informed consent for inclusion within the study. Those that lack capacity were enrolled via consultee declaration (from family members/next of kin or from professionals if no family were available).

## Funding

The author’s role is funded by the Mental Health and Neurosciences Doctoral Training Programme (Wellcome Trust) (Grant No. MHN DTP-223508/Z/21/Z).

The funder had no input to the design of output of the referenced study.

## Declaration of Generative AI and AI-assisted technologies in the writing process

Generative AI, ChatGPT 5.2, was used in the production of this manuscript to ensure alignment with US punctuation and spelling conventions. The prompt used was, “Review this manuscript and highlight areas where non-US spelling, grammar and punctuation have been used.” All recommended changes were adopted. The author retains full accountability for the generated outputs.

## CRediT authorship contribution statement

**David Wenzel:** Writing – original draft, Validation, Project administration, Investigation, Funding acquisition, Formal analysis, Data curation, Conceptualization.

## Declaration of Competing Interests

The authors declare that they have no known competing financial interests or personal relationships that could have appeared to influence the work reported in this paper.

## Footnotes

1 Pseudonyms are used throughout this commentary.
